# Assessing the Long‐Term Survival of Dental Implants in A Retrospective Analysis: Immediate Versus Delayed Placement

**DOI:** 10.1002/cre2.70096

**Published:** 2025-03-06

**Authors:** Georgios S. Chatzopoulos, Larry F. Wolff

**Affiliations:** ^1^ Division of Periodontology University of Minnesota Minneapolis Minnesota USA; ^2^ Faculty of Dentistry, Health Sciences Aristotle University of Thessaloniki Thessaloniki Greece

## Abstract

**Objectives:**

This large‐scale retrospective study aims to evaluate and compare the long‐term survival rates of dental implants placed immediately after tooth extraction (type 1) versus those placed at a later stage (types 2, 3, and 4). Additionally, it examines how patient characteristics and implant site conditions influence the choice of implant placement.

**Materials and Methods:**

This study retrospectively analyzed patient data from 10 university dental clinics between 2011 and 2022 and examined dental implant treatment outcomes. Patient information, including age, sex, ethnicity, race, smoking, and medical status, was analyzed.

**Results:**

Records of 20,842 patients with 50,333 dental implants inserted between 2011 and 2022 were analyzed. The multivariate analysis resulted in significant differences for age, ethnicity, race, gender, and asthma. A 98.4% survival rate for dental implants placed immediately following extraction and a 98.6% survival rate for those placed in fully healed sockets were recorded. The type of implant placement (immediate vs. delayed) showed no significant effect on implant outcome.

**Conclusion:**

Immediate implant placement resulted in high survival rates with delayed implants inserted into healed sites. Both immediate and delayed implant placements are viable therapeutic approaches demonstrating predictable outcomes.

## 1 Introduction

Dental implants have revolutionized dentistry and implant therapy has become a widely acceptable treatment modality for missing teeth after its introduction in the 1970s by Branemark (Brånemark et al. 1977). Studies have shown favorable survival and success rates for implants in the general population in the long term (Lang et al. 2004; Pjetursson et al. 2004). Increased implant survival has been recorded in various clinical scenarios and patient populations (De Boever et al. 2009; Bassir et al. 2019; Derks et al. 2015; Salinas and Eckert 2007). However, specific factors may increase an individual's likelihood of experiencing lower success rates and an increased failure risk (Sakka and Coulthard 2011). Understanding the factors that contribute to implant failure is crucial for both clinicians and patients to make informed decisions and achieve optimal long‐term outcomes. Implant failure refers to the inability of a dental implant to integrate successfully with the surrounding bone or maintain its stability over time. This may be attributed to infection, inadequate bone support, poor oral hygiene, systemic conditions, and mechanical overload (Sakka and Coulthard 2011). Although implant success rates have steadily improved, research suggests that the timing of implant placement may influence the risk of failure (Carosi et al. 2023). Immediate implant placement involves inserting the implant directly into the extraction socket after tooth removal (type 1) (Chen and Buser 2009). Early implant placement occurs within 4–8 weeks after extraction, delayed implant placement is performed 12 weeks or more after extraction, and late/conventional placement is considered when an implant is inserted at least 4 months following the extraction (Chen and Buser 2009). Each timing has its advantages and disadvantages, with varying impacts on implant survival rates.

Immediately placed implants offer advantages such as lower treatment time and bone preservation and soft tissue architecture, which may lead to better aesthetic treatment outcomes (Liñares et al. 2023; Donos et al. 2021; Garcia‐Sanchez et al. 2022; Lang, Pun, Lau, Li, et al. 2012). In addition, it can lead to higher patient satisfaction than the conventional treatment approach due to the decreased number of surgeries and the reduced discomfort (Schropp and Isidor 2008). Immediate implant placement, while offering potential benefits, also presents several disadvantages and risks, including poor aesthetic outcomes due to the unpredictable soft tissue healing, reduced primary stability, and the potential need of additional procedures such as grafting, and it is also considered a technically demanding procedure (Yuenyongorarn et al. 2020; Romanos 2015; Esposito et al. 2010; Ardekian and Dodson 2003).

A comprehensive review of the literature reported that although both immediate and delayed implants have high survival rates, immediate placement may lead to a slightly higher risk of complications, including early failure (Chen and Buser 2009). A recent meta‐analysis that compared the survival of implants placed immediately and delayed following tooth extraction reported no significant differences, whereas a focused analysis showed that slightly more failures occurred in the immediate dental implant placement group (Patel et al. 2023). Another meta‐analysis showed that immediate implants placed in fresh extraction sockets show significantly lower survival rates than delayed implants, whereas no differences were detected in terms of marginal bone loss, implant stability, and probing pocket depths (Mello et al. 2017). Moreover, a meta‐analysis that included only randomized clinical trials concluded that immediate implants in the anterior area showed better results, whereas molar sites showed more favorable outcomes with delayed implants (Canellas et al. 2019).

The timing of implant placement is a complex factor influencing implant treatment outcome and the optimal timing varies depending on individual patient factors. Although some studies suggest a higher risk of failure with immediate implant placement, others report comparable rates with delayed placement. However, long‐term and large‐scale studies are needed that will lead to strong conclusions. Therefore, the present large‐scale retrospective study aims to evaluate and compare the long‐term survival rates of dental implants placed immediately after tooth extraction (type 1) versus those placed at a later stage (types 2, 3, and 4). Additionally, it examines how patient characteristics and implant site conditions influence the choice between immediate and delayed implant placement.

## 2 Materials and Methods

This study retrospectively analyzed patient data from university dental clinics within the BigMouth network. Data were collected between 2011 and 2022 and dental implant treatment outcomes were examined. Institutional review board (IRB) review waiver was granted for this study. This study adhered to the ethical principles of the Helsinki Declaration. Informed consent was not needed due to the retrospective study design.

### 2.1 Study Population

Patient records from university dental schools within the BigMouth network between 2011 and 2022 were examined to assess dental implant treatment outcomes. Patients who had undergone implant placement were identified using specific dental procedure codes, CDT D6010 (implant placement). Implant failure was determined based on relevant procedure codes indicating implant removal, CDT D6100 (implant removal). All examined patients should have received at least one of the following: comprehensive oral evaluation (D0150), periodic oral evaluation (D0120), or comprehensive restorative and periodontal exam (D0180).

Individuals who visited any of the included university dental clinics and had undergone implant treatment were identified. Records of patients with complete demographic and medical history information were retrieved. The examined systemic medical conditions were self‐reported.

### 2.2 Data Extraction

Patient data, including age, sex, ethnicity, race, smoking, and medical history, were collected from electronic health records, organized into a new data set, and then validated by data analysts. This information was used to examine potential factors influencing implant outcomes, considering variables such as age at implant placement, ethnicity, sex, race, tobacco use, and the presence or absence of specific medical conditions, including cardiovascular disorders, endocrine disorders, infectious diseases, kidney disorders, musculoskeletal disorders, neurological disorders, and respiratory disorders.

### 2.3 Implant Failure and Survival Definitions

Implants removed for any reason were considered “failures” as assessed during the patient's latest follow‐up visit. On the other hand, implants remaining in function to support a restoration, without any need for removal at the last recall appointment, were considered “survivals.” The overall outcome of the treatment was then classified as either survival or failure.

### 2.4 Statistical Analysis

Patient and implant characteristics were presented as frequencies, means, and standard deviations. *χ* ^2^ and *t*‐tests were used to compare immediate and delayed implant placement groups. Patient‐related and implant site‐related variables found to be significant in univariate analysis (*p* < 0.05) were subsequently introduced into a multivariable regression model. The 95% confidence interval (CI) and odds ratio (OR) were calculated and reported. Survival analysis was performed at implant‐level data using Kaplan–Meier analysis. Cox regression models analyzed time to failure, comparing immediate and delayed implant treatments. Patients without implant failure were censored at their last follow‐up. An adjusted model examined the impact of various factors on implant survival, reporting hazard ratios and 95% CIs for each model. A statistical software program was used for analysis (SPSS v.29.0, IBM, Armonk, NY, USA). The level of significance was 0.05.

## 3 Results

### 3.1 Patient‐Related Outcomes

Records of implants inserted in 20,842 patients between 2011 and 2022 were analyzed. The majority of the included patients (86.9%) had a dental implant placed following the delayed approach. The characteristics of patients who received immediate or delayed implants, as well as the entire patient group involved in the study, are shown in Table 1. Significant differences between the immediate and delayed implant patient groups were detected in terms of age, gender, ethnicity, race, arthritis, and asthma. Patients who received a dental implant immediately following tooth extractions were significantly older, more likely females, and self‐reported more frequently a diagnosis of arthritis and asthma than those who had a delayed implant. Hispanic ethnic group patients and African‐Americans more often received delayed rather than immediate implants. The type of implant placement (immediate vs. delayed) showed no significant effect on treatment outcome (*p* = 0.28).

**Table 1:** Patient‐related characteristics of the immediate and delayed implant treatment groups as well as of the total population.

Patient‐related characteristics	Total population *N* = 20,842—100%	Immediate implant patient group *n* = 2734—13.1%	Delayed implant patient group *n* = 18,108—86.9%	*p*‐value^a^
Age (mean (SD))	57.50 (14.27)	59.43 (13.68)	57.21 (14.33)	**< 0.001**
Gender (%)				
Female	10,798 (51.8)	1470 (53.8)	9,328 (51.5)	**0.03**
Male	10,041 (48.2)	1264 (46.2)	8777 (48.5)	
Ethnicity (%)				
Non‐Hispanic	18,994 (91.1)	2,518 (92.1)	16,476 (91.0)	**< 0.001**
Hispanic	1294 (6.2)	126 (4.6)	1168 (6.5)	
Others	554 (2.7)	90 (3.3)	464 (2.5)	
Race (%)				
White	9,900 (66.3)	1,399 (68.5)	8,501 (65.9)	**0.05**
Asian	935 (6.3)	120 (5.9)	815 (6.3)	
African American	962 (6.4)	100 (4.9)	862 (6.7)	
Hispanic or Latino	1173 (7.9)	158 (7.7)	1,015 (7.9)	
Pacific Islander	65 (0.4)	6 (0.3)	59 (0.5)	
American Indian or Alaskan Native	59 (0.4)	7 (0.3)	52 (0.4)	
Others	1843 (12.3)	252 (12.3)	1591 (12.3)	
Tobacco use (%)	1673 (8.0)	221 (8.1)	1452 (8.0)	0.91
Hypertension (%)	3393 (16.3)	443 (16.2)	2950 (16.3)	0.93
Marijuana use (%)	218 (1.0)	30 (1.1)	188 (1.0)	0.77
Diabetes (%)	1198 (5.7)	178 (6.5)	1020 (5.6)	0.07
Thyroid disorder (%)	1156 (5.5)	171 (6.3)	985 (4.7)	0.09
HIV (%)	62 (0.3)	5 (0.2)	57 (0.3)	0.34
Kidney disease (%)	820 (3.9)	122 (4.5)	698 (3.9)	0.12
Arthritis (%)	2061 (9.9)	308 (11.3)	1753 (9.7)	**0.01**
Osteoporosis (%)	621 (3.0)	93 (3.4)	528 (2.9)	0.16
Depression (%)	1226 (5.9)	172 (6.3)	1054 (5.8)	0.34
Seizures/Epilepsy (%)	93 (0.4)	10 (0.4)	83 (0.5)	0.64
Asthma (%)	874 (4.2)	162 (5.9)	712 (3.9)	**< 0.001**
Sleep apnea (%)	442 (2.1)	59 (2.2)	383 (2.1)	0.89
Treatment outcome				
Survived	20,274 (97.3)	2651 (97.0)	17,623 (97.3)	0.28
Failed	568 (2.7)	83 (3.0)	485 (2.7)	

Abbreviation: SD, standard deviation

a Statistical significance with *p*‐value ≤ 0.05 is shown in bold.

The multivariate analysis resulted in significant differences for age (*p* < 0.001), ethnicity (*p* < 0.001), race (*p* = 0.01), gender (*p* = 0.03), and asthma (*p* < 0.001). Asthmatic patients were significantly more likely to receive immediate than delayed implants (1.45, 1.20–1.75, *p* < 0.001). Male patients (1.11, 1.01–1.22, *p* = 0.03), Asians (1.42, 1.06–1.91, *p* = 0.02), African‐Americans (1.84, 1.36–2.49, *p* < 0.001), White patients (1.41, 1.13–1.77, *p* < 0.001), and individuals of some other races (1.48, 1.13–1.93, *p* = 0.01) received a delayed implant significantly more frequently. Similarly, Hispanics (2.29, 1.60–3.29, *p* < 0.001) and non‐Hispanics (1.33, 1.02–1.74, *p* = 0.04) were significantly more likely to receive an implant following the delayed approach.

### 3.2 Implant Site‐Related Outcomes

The characteristics specific to the implant site, including details about the location and surrounding conditions, are shown for both groups and the entire study population in Table 2. A total of 50,333 implant records were included in the analysis; the majority of them (*n* = 46,353, 92.1%) were records of implants placed after tooth extraction (delayed implant placement). In total, 725 failures occurred from the 50,333 included implants, leading to a survival rate of 98.6% in 83.86 ± 57.57 months (range: 0–367 months). Timing of implant placement (immediate and delayed) was significantly associated with the implant's location (*p* < 0.001). Maxilla and anterior sites received immediate implants significantly more often than delayed implants (*p* < 0.001). The implant placement type was found to have no significant association with the treatment outcome (*p* = 0.16). The multivariate analysis revealed that delayed implants were significantly more likely to be placed in the mandible (OR: 1.33, 95% CI: 1.24–1.42, *p* < 0.001) than in the maxilla. In addition, posterior implants were significantly more frequently replaced by implants following a delayed approach (OR: 2.87, 95% CI: 2.69–3.07, *p* < 0.001) than immediate ones.

**Table 2:** Implant site‐related characteristics of the immediate and delayed implant treatment groups as well as of the total population.

Implant site‐related characteristics	Total population, *N* = 50,333—100%	Immediate implant group, *n* = 3980 —7.9%	Delayed implant group, *n* = 46,353 —92.1%	*p*‐value^a^
Jaw (%)				
Maxilla	26,918 (53.5)	2462 (61.9)	24,256 (52.8)	**< 0.001**
Mandible	23,415 (46.5)	1518 (38.1)	21,897 (47.2)	
Region (%)				
Posterior	36,164 (71.8)	1946 (48.9)	34,218 (73.8)	**< 0.001**
Anterior	14,169 (28.2)	2034 (51.1)	12,135 (26.2)	
Treatment outcome				
Survived	49,608 (98.6)	3915 (98.4)	45,693 (98.6)	0.16
Failed	725 (1.4)	65 (1.6)	660 (1.4)	

Abbreviation: SD, standard deviation.

a Statistical significance with *p*‐value ≤ 0.05 is shown in bold.

### 3.3 Survival Analysis

**Figure 1:**
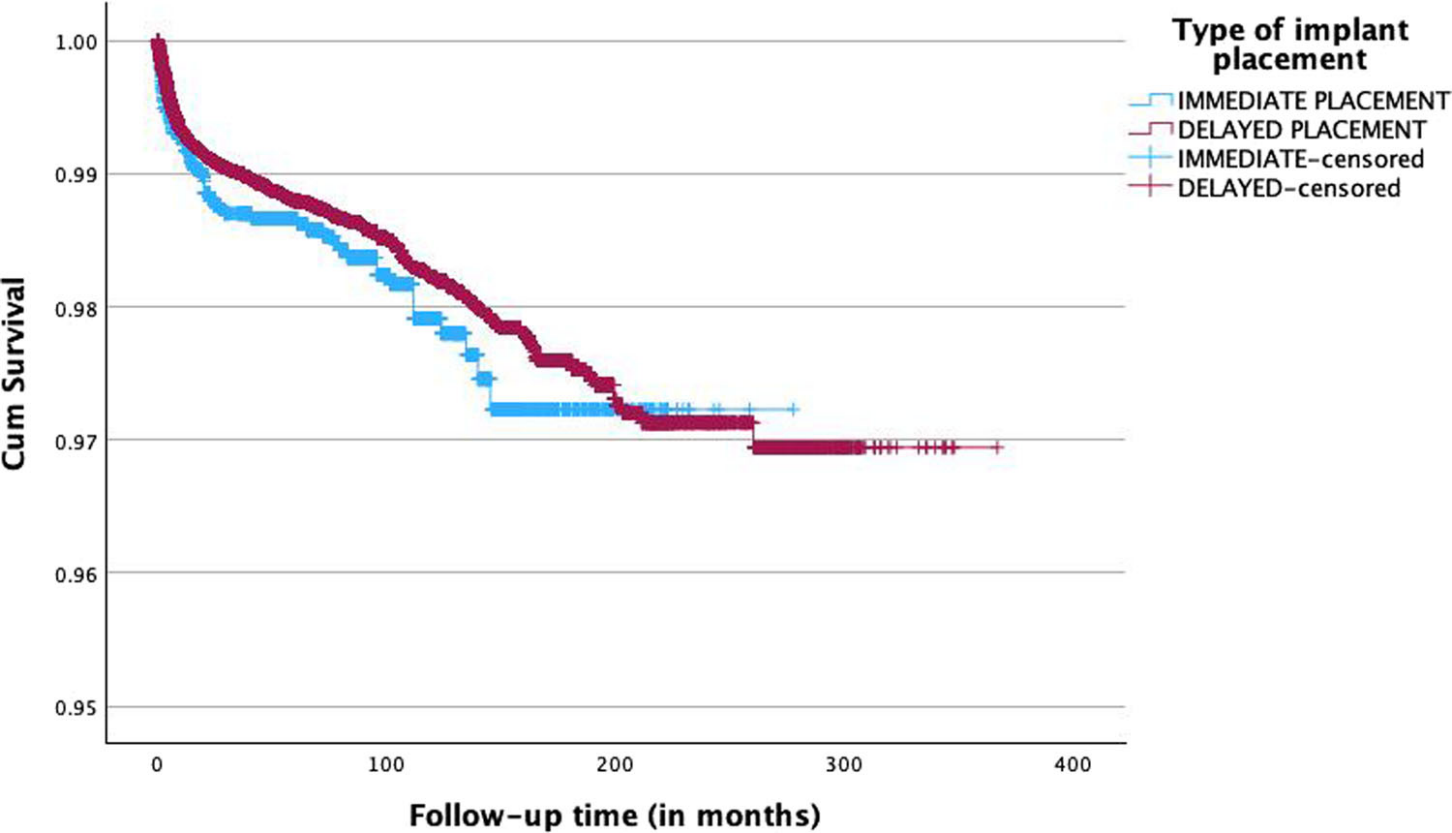
Cumulative survival rates of immediate and delayed implants with respect to time (in months).

The survival rates of implants placed immediately and in a delayed mode following tooth extraction in terms of time (in months) are shown in Figure 1. The survival rate of the immediate implants was 98.4% (65 failures of the total 3980 implants), whereas delayed implants showed a 98.6% rate of survival (660 failed implants of the total 46,353). The estimated mean survival time for immediate implants was 271.74 (270.17–273.32) months, whereas delayed implants placed in healed sockets showed a mean survival time of 358.47 months (357.58–359.37) months. The overall mean survival time was 358.41 (357.53–359.28) months. The mean survival time was similar between the immediate and delayed implants (*p* = 0.19). Almost a third of the included immediate implants (21 out of 65, 32.31%) failed within the first 4 months of healing. During the same healing time, in the delayed implant group, only 149 failures occurred out of the 660 recorded (22.58%). The multivariable Cox regression model depicted no significant associations between the jaw (*p* = 0.60) and the region (*p* = 0.17) with the implant treatment outcome.

## 4 Discussion

This retrospective investigation aimed to assess and compare the survival rates of implants placed immediately and delayed following tooth extraction and identify the factors related to therapy selection utilizing a large dental records database between 2011 and 2022. These were analyzed and the main findings were as follows:

- A 98.4% survival rate for dental implants placed immediately after tooth extraction (type 1) and a 98.6% survival rate for those placed in healed sockets (type 2, 3, and 4) were recorded. However, these results might be slightly influenced by differences in baseline characteristics between the two groups, such as patient‐related variables, including age, gender, ethnicity, race, arthritis, and asthma, as well as implant site‐related factors, including the region and the jaw at which an implant was placed.
- The implant treatment outcome was not related to the type of implant placement.
- Maxilla and anterior sites received immediate implants significantly more often than delayed implants.
- Asthmatic patients were significantly more likely to receive immediate than delayed implant, whereas implants following a delayed treatment approach were more often placed in male patients, individuals of specific race groups, including Asians, African‐Americans, White patients, and individuals of other races. Similarly, Hispanic and non‐Hispanic ethnic groups received delayed implants more often.

The ideal timing for dental implant placement, either immediately after tooth extraction or delayed until tissue healing, remains a subject of debate within implant dentistry. Conflicting evidence exists regarding the outcomes of each approach. Although some studies indicate no significant differences in failure rates, complications, or patient satisfaction between immediate and delayed placement, others show that immediate implants may lead to greater bone loss and less favorable aesthetic outcomes (Felice et al. 2016; Tonetti et al. 2017). Additionally, a prospective multicenter clinical study with a 3‐year follow‐up of 264 implants showed similar cumulative survival rates for both immediate and delayed implants (Grunder et al. 1999). Similar findings were observed for implants in the anterior and maxillary molar region, with implants placed immediately or after socket healing (Palattella et al. 2008; Peñarrocha‐Oltra et al. 2012). Whether dental implants were placed immediately or after a delay had no impact on their long‐term success rates, regardless of implant shape (tapered or cylindrical) or when they were assessed within 5 years of being loaded (Zafiropoulos et al. 2010). Our findings align with these investigations.

Systematic reviews and meta‐analyses have further explored this topic, revealing contrasting results. Some suggest higher failure rates for immediate implants, urging caution in their use, whereas others highlight high success rates and patient satisfaction with limited complications. Chen and Buser's early systematic review on immediate, early, and delayed implant placements showed promising survival rates exceeding 95% for most studies, with comparable outcomes for immediate and early placement (Chen and Buser 2009). In another systematic review evaluating survival rates of immediate implants, 46 prospective studies resulted in a survival rate of 98.4% and the authors highlighted the impact of antibiotic regimens on implant success, emphasizing the importance of postsurgical antibiotics (Lang, Pun, Lau, et al.^2012^). Specifically, the annual failure rate was higher (1.78%) for patients who received only a single preoperative antibiotic dose compared with those who received a 5–7‐day postoperative course (0.51%) or a single preoperative and 5–7‐day postoperative course (0.75%), regardless of the antibiotic used (Lang, Pun, Lau, et al. 2012). In the present investigation, no information was available with respect to the antibiotic regimen used.

Several studies have investigated the survival rates of immediate versus delayed implants. Some reviews, like those by Mello and colleagues, found that immediate implants had a higher failure rate and advised caution (Mello et al. 2017). Another meta‐analysis also noted lower survival for immediate placement but no differences in other clinical parameters (Cosyn et al. 2019). However, immediate implants have shown high success rates with good patient satisfaction (Thanissorn et al. 2022). A large meta‐analysis of 17,278 immediate implants and 38,738 delayed implants also indicated a greater failure risk for immediate implants, primarily in the maxilla (Ibrahim and Chrcanovic 2021).

Research on risk factors influencing immediate implant failure has yielded mixed results. One study found that patients not using amoxicillin were more prone to implant failure and showed 3.34 times higher risk of experiencing implant failure (Wagenberg and Froum 2006). This could not be assessed in the present investigation, as no such information was available. Although periodontitis has been associated with an increased risk of implant failure (Serroni et al. 2024), systematic reviews have not reported an increased risk for individuals with a history of periodontitis in immediately placed implants (Lang, Pun, Lau, et al. 2012; Kaur et al. 2021). The periodontal status of the included population was not known in the present study and therefore this parameter could not be assessed. Moreover, implant location (anterior vs. posterior, maxillary vs. mandibular) did not significantly affect survival rates. In agreement with our findings, implant survival rates did not differ significantly between maxillary and mandibular or anterior and posterior placements in a systematic review that included 46 studies with 967 implants in the posterior area and 487 in the anterior area (Lang, Pun, Lau, et al.^2012^). Although the estimated annual failure rate was higher for posterior implants, this difference was not statistically significant (Lang, Pun, Lau, et al.^2012^). Similarly, although maxillary implants showed a slightly higher annual failure rate than mandibular implants, the difference was not statistically significant (Lang, Pun, Lau, et al.^2012^).

Apical periodontitis may pose an increased risk for implant failure, especially in immediate implants. Early studies cautioned against immediate placement in sites with infection due to the risk of microbial contamination (Quirynen et al. 2003; Rosenquist and Grenthe 1996). However, recent evidence suggests that with thorough cleaning, debridement, and chlorhexidine rinse before placement, immediate implants in such sites can achieve comparable outcomes to those in healthy sites (Chrcanovic et al. 2015; Crespi et al. 2017; Lee et al. 2015). Unfortunately, due to the retrospective design of this study, information regarding apical periodontitis and its impact on implant outcomes was unavailable for the included patients. In addition, it is worth mentioning that it was not within the scope of the present investigation to report relationships between implant failure and patient‐related or implant site‐related factors. In a study published by our group and that included the same 20,842 patients who received 50,333 dental implants, it was demonstrated that ethnicity and race were significantly associated with implant failure (Chatzopoulos and Wolff 2023). Both Hispanic and non‐Hispanic ethnic groups, along with White and African American individuals, showed a notably higher risk of experiencing dental implant failure compared with patients of Hispanic descent (Chatzopoulos and Wolff 2023). In addition, implant failure was not significantly associated with systemic medical conditions (Chatzopoulos and Wolff 2023).

The retrospective nature of this study, with its varied treatment clinicians and absence of information on periodontal condition and bone augmentation procedures, could potentially introduce biases. However, previous research suggests that implant survival rates are comparable between native and bone‐grafted sites (Tran et al. 2016), mitigating some concerns related to the lack of bone augmentation data. Furthermore, due to the study's retrospective design and reliance on electronic records, it was not feasible to assess the success rates through clinical and radiographic examinations.

This study has several limitations that warrant consideration. Primarily, the retrospective nature of our analysis and reliance on records restricted our ability to comprehensively evaluate implant success. Although the assessment of implant survival was defined as the presence or absence of the implant, the data lacked consistent and detailed information on key parameters such as probing depths, bleeding on probing, radiographic bone loss, and technical complications such as screw loosening, screw fracture, and porcelain chipping. These parameters are essential for a thorough evaluation of implant success. Consequently, our findings primarily reflect implant survival and may not fully capture the long‐term functional and biological success of implants. In addition, due to the retrospective nature of our analysis and reliance on existing records, we were unable to consistently obtain detailed information on implant types, grafting materials, loading protocols, and types of prostheses. This limitation may restrict the generalizability of our findings, as the specific characteristics of implants and treatment protocols can influence outcomes. Future prospective studies with standardized clinical and radiographic assessments as well as data collection methods are needed to provide a more comprehensive evaluation of implant success following immediate and delayed implant placement.

A notable advantage of this study is the assessment of a substantial volume of dental implant records from 10 university clinics in the United States, where treatments adhered to evidence‐based protocols, enhancing the validity of our findings and minimizing selection bias. However, to fully understand implant performance in real‐world settings, additional data from everyday dental practices are necessary. Another significant advantage of this study is its extensive follow‐up period, tracking implants for up to 367 months, which surpasses the duration of many previous investigations in this field. Future prospective studies should investigate the success rates of various types of implant placements, along with their associated risk factors, to enable personalized treatment plans and optimize patient outcomes.

## 5 Conclusion

Within the limitations of this large‐scale, record‐based, retrospective study, which analyzed implant records of 10 university dental clinics in the United States, dental implants placed immediately after extraction (type 1) had a comparable survival rate (98.4%) to those placed in healed sockets‐ types 2, 3, and 4 (98.6%). Prospective studies are necessary to provide a more comprehensive evaluation of implant success following immediate and delayed implant placement.

## Author Contributions

Conceptualization, acquisition of data, and design: Georgios S. Chatzopoulos and Larry F. Wolff. Analysis, interpretation of results, and drafting of the manuscript: Georgios S. Chatzopoulos. Critical review of the manuscript: Larry F. Wolff. All authors approved the final version of the manuscript.

## Conflicts of Interest

The authors declare no conflicts of interest.

## Data Availability

The data of the study are available upon reasonable request.
